# Electrical stimulation shifts healing/scarring towards regeneration in a rat limb amputation model

**DOI:** 10.1038/s41598-019-47389-w

**Published:** 2019-08-07

**Authors:** K. M. C. Oliveira, J. H. Barker, E. Berezikov, L. Pindur, S. Kynigopoulos, M. Eischen-Loges, Z. Han, M. B. Bhavsar, D. Henrich, L. Leppik

**Affiliations:** 1 Frankfurt Initiative for Regenerative Medicine, Experimental Orthopedics & Trauma Surgery, J.W. Goethe University, Frankfurt am Main, Germany; 2 European Research Institute for the Biology of Ageing, University Medical Center Groningen, Groningen, The Netherlands; 3 Present Address: Department of Plastic, Hand and Reconstructive Surgery, BG Trauma Center Frankfurt am Main gGmbH, Frankfurt am Main, Germany; 4 Department of Trauma, Hand and Reconstructive Surgery, J.W. Goethe University, Frankfurt am Main, Germany

**Keywords:** Morphogenesis, Developmental biology

## Abstract

Different species respond differently to severe injury, such as limb loss. In species that regenerate, limb loss is met with complete restoration of the limbs’ form and function, whereas in mammals the amputated limb’s stump heals and scars. In *in vitro* studies, electrical stimulation (EStim) has been shown to promote cell migration, and osteo- and chondrogenesis. In *in vivo* studies, after limb amputation, EStim causes significant new bone, cartilage and vessel growth. Here, in a rat model, the stumps of amputated rat limbs were exposed to EStim, and we measured extracellular matrix (ECM) deposition, macrophage distribution, cell proliferation and gene expression changes at early (3 and 7 days) and later stages (28 days). We found that EStim caused differences in ECM deposition, with less condensed collagen fibrils, and modified macrophage response by changing M1 to M2 macrophage ratio. The number of proliferating cells was increased in EStim treated stumps 7 days after amputation, and transcriptome data strongly supported our histological findings, with activated gene pathways known to play key roles in embryonic development and regeneration. In conclusion, our findings support the hypothesis that EStim shifts injury response from healing/scarring towards regeneration. A better understanding of if and how EStim controls these changes, could lead to strategies that replace scarring with regeneration.

## Introduction

Limb regeneration – an organisms’ ability to regrow a healthy normally functioning limb - has intrigued scientists for ages (reviewed in^1^). It is well known that some vertebrates, such as salamanders, frogs and zebra fish are able to regrow partial or complete appendages and organs^2^, however, mammals’ endogenous ability to regenerate is limited to a few exceptions like the terminal phalanges in marsupials and rodents, and distal fingertips in young children (reviewed in^3,4^). Most adult human tissues do not regenerate, instead, after injury, healing occurs governed by a fibro proliferative response leading to scar tissue formation (reviewed in^5^). Whether and to what degree a tissue “heals and scars” versus “regenerates” depends on the tissues’ and organs’ inherent regenerative ability, the type and extent of the lesion, and local and systemic physical and chemical signals that regulate these processes (reviewed in^6^).

In response to injury both non-regenerative and regenerative amputation wounds undergo distinct overlapping cascades of events in which hemostasis and inflammatory response occur first, in both (reviewed in^7^). During the inflammatory phase of healing, damaged and dead cells are phagocytized, and cell migration and mitosis are induced by platelet-derived growth factors (reviewed in^8^). In case of regenerative response, the amputation site is invaded by macrophages as well as pro- and anti-inflammatory cytokines at the same time. In contrast in regeneration-incompetent wounds, the pro-inflammatory response occurs first and is then followed by the anti-inflammatory response^9,10^. Additionally, cellular proliferation is essential in early phases of regeneration, in which case epithelial cells cover the surface of wounds in the first 24 hours, with an epidermal layer of cells^11^.

In healing/scarring injury response, following hemostasis and the inflammation phases, proliferation takes over in which case collagen deposition, new vascularization and an increase in fibroblast activity occur (reviewed in^12^). The deposition of collagen and fibronectin form a provisional extracellular matrix (ECM), and proliferating epithelial cells cover the wounded tissues (reviewed in^13^). Besides providing structural support to which migrating cells adhere, ECM facilitates cell growth during homeostasis and tissue repair (reviewed in^14^). In regenerative injury response, progenitor cells are activated and accumulate beneath the newly created epithelial cell layer forming a blastema (reviewed in^15^). To promote successful appendage regeneration, provisional ECM, deposited during blastema formation, is rich in collagen type III and subpopulations of fibroblasts^16^. The ECM deposited in the blastema contains patterning information for the regrowing amputated part^17^. Once the blastema reaches a critical size, it flattens and cells begin to differentiate and patterning begins to form the new structures (reviewed in^18^). In contrast in non-regenerative amputation wounds a blastema is not formed, instead the tissues undergo maturation and remodeling, whereby collagen is restructured along tension lines to form scar tissue and nonessential cells experience apoptosis (reviewed in^8^).

Recent literature indicates that bioelectric signaling may play an important role in both healing/scarring and regeneration processes^19,20^. For example, mammals’ wounds display a positive polarity throughout the healing process, whereas in regenerating amphibians the polarity is initially positive and quickly changes to negative with the peak voltage occurring at the time of maximum cellular proliferation (reviewed in^21^). Intrigued by the possibility of recreating this bioelectric environment, seen in regenerating amphibians, researchers have tried manipulating bioelectric fields to induce regeneration in adult mammal tissues. As early as 1972 Becker and Spadaro hypothesized that changing wound polarity by applying exogenous electrical stimulation (EStim) in mammals could improve wound healing and perhaps even induce a regenerative response^22^. In his landmark article, published in 1972 in the journal Nature, Becker reported “blastema formation, new bone, bone marrow, cartilage, nerve, skin, muscle, and epiphyseal plate formation” in amputated rat limbs treated with low voltage direct current electrical stimulation. Based on these findings he concluded “regenerative growth can be restored in mammals by application of the appropriate levels of electrical stimulations”^23^.

Since then, several studies have shown that EStim can affect the healing process (reviewed in^24^). Jang *et al*., demonstrated that the use of external EStim enhanced regenerative activities at different stages of wound healing. They reported up-regulation of inflammatory macrophages and vascular endothelial growth factor (VEGF) in the inflammatory phase; increased cell proliferation markers and protein expression of type III collagen, TGF-β, Integrin α5 and *Mmp2* in the proliferative phase; and increased expression of fibronectin and types I and IV collagen during the remodeling phase^25^. Supporting these findings, *in vitro* studies demonstrate that EStim influences cellular functions, such as migration, proliferation and differentiation, that play a central role in regeneration^26–31^.

In recent *in vivo* studies we reproduced Becker’s original experiment and while we did not observe blastema formation we did demonstrate that low voltage direct current EStim treatment induced significant growth of bone, cartilage and vessels in the same amputated rat limb model^32^. Having shown this positive effect of EStim, we then questioned if EStim modified important mechanisms, after injury, that support regeneration, which in turn suppress scar formation. In other words, we asked if EStim shifted the balance in the injury response away from healing/scarring towards regeneration. To answer this, we conducted a series of studies in the same rat limb amputation model in which we investigated the effects of EStim on ECM deposition, macrophage distribution, cell proliferation and gene expression changes in EStim treated and non-treated rat limb stump tissues, in early (3 and 7 days) and later stages (28 days) after amputation.

## Results

### Collagen/Extracellular matrix deposition

Extracellular matrix (ECM) remodeling, i.e. ECM degradation, synthesis, reassembly and modification, is a critical distinguishing characteristic between tissue healing/scarring and regeneration. We evaluated differences in collagen fibril distribution in stump tissue at 3-, 7- and 28- days post amputation by measuring fibril orientation (anisotropy) and the distance between fibrils (condensation).

**Figure 1:**
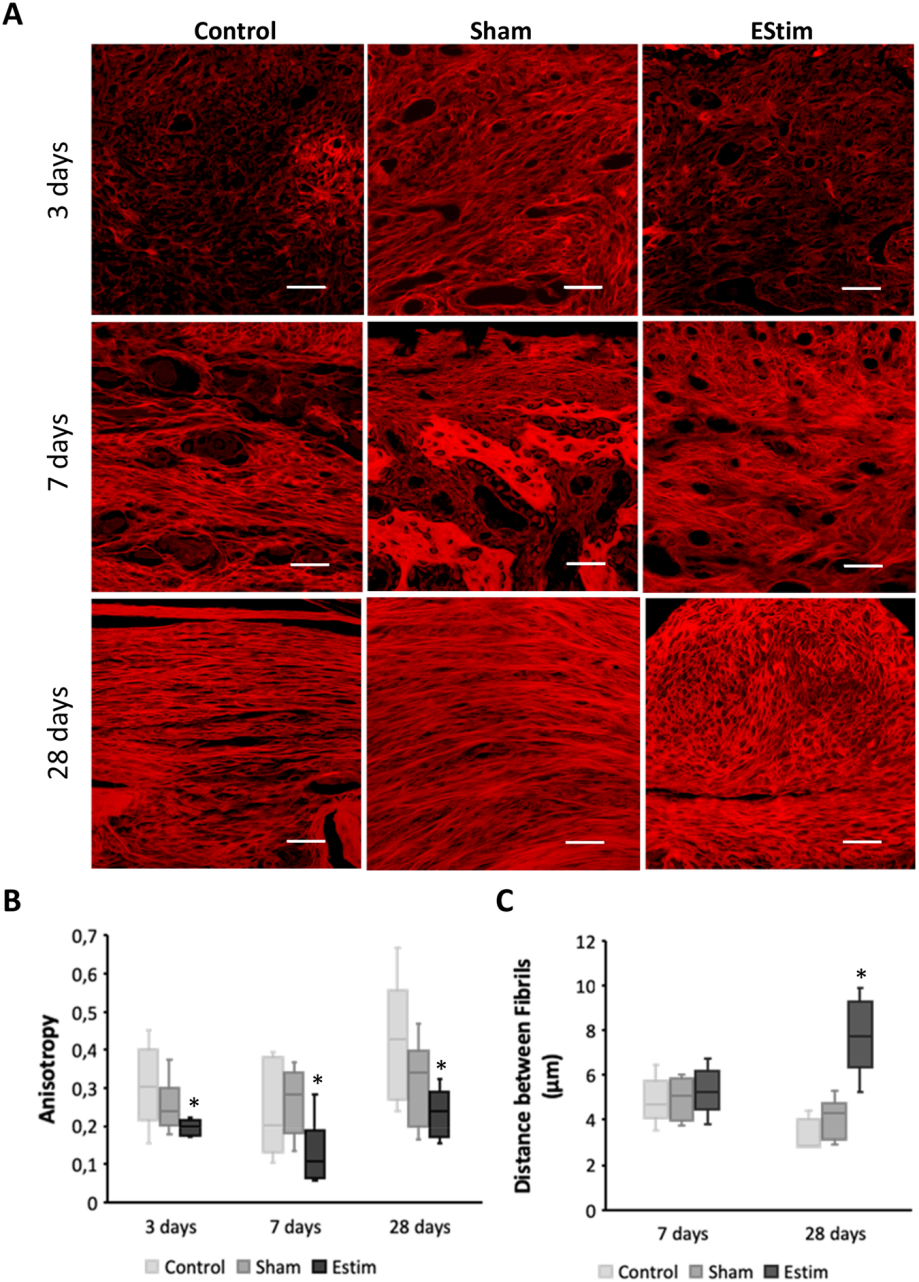
Extracellular matrix (ECM) deposition at the distal end of limb stumps for all groups at different time points. (**A**) Representative images of Picro Sirius Red stained collagen network at the distal end of limb stumps for control, sham and EStim groups at 3, 7, and 28 days post amputation (Scale bar = 100 µm). (**B)** Graph showing lower anisotropy (parallelism between fibrils) in electrically stimulated stump tissue compared to non-treated stumps, at all the time points evaluated. (**C**) Graph showing distance between fibrils measured at days 7 and 28 post-amputation. The greatest distance between fibrils was shown in EStim treated tissue at day 28. *p ≤ 0.05.

As shown in Fig. 1 there were important differences in fibril orientation between the EStim treated, control and sham stump tissues.

Whereas in sham and control stumps fibers appear tightly packed into parallel fibril layers over the stump, in EStim treated stumps fibrils appear less organized and distributed haphazardly with more space in-between fibrils (Fig. 1A). This difference in ECM deposition is evident in the stump tissues as early as 3 days post amputation. Quantification of collagen fibril parallelism (anisotropy) confirmed that EStim significantly (p < 0.05) decreased fibril anisotropy, compared to sham and control stump tissues (Fig. 1B). Moreover, the distance between fibrils was significantly (p < 0.05) greater in EStim treated stump tissues compared to both control groups at 28 days post amputation (Fig. 1C).

### Macrophage number and distribution

**Figure 2:**
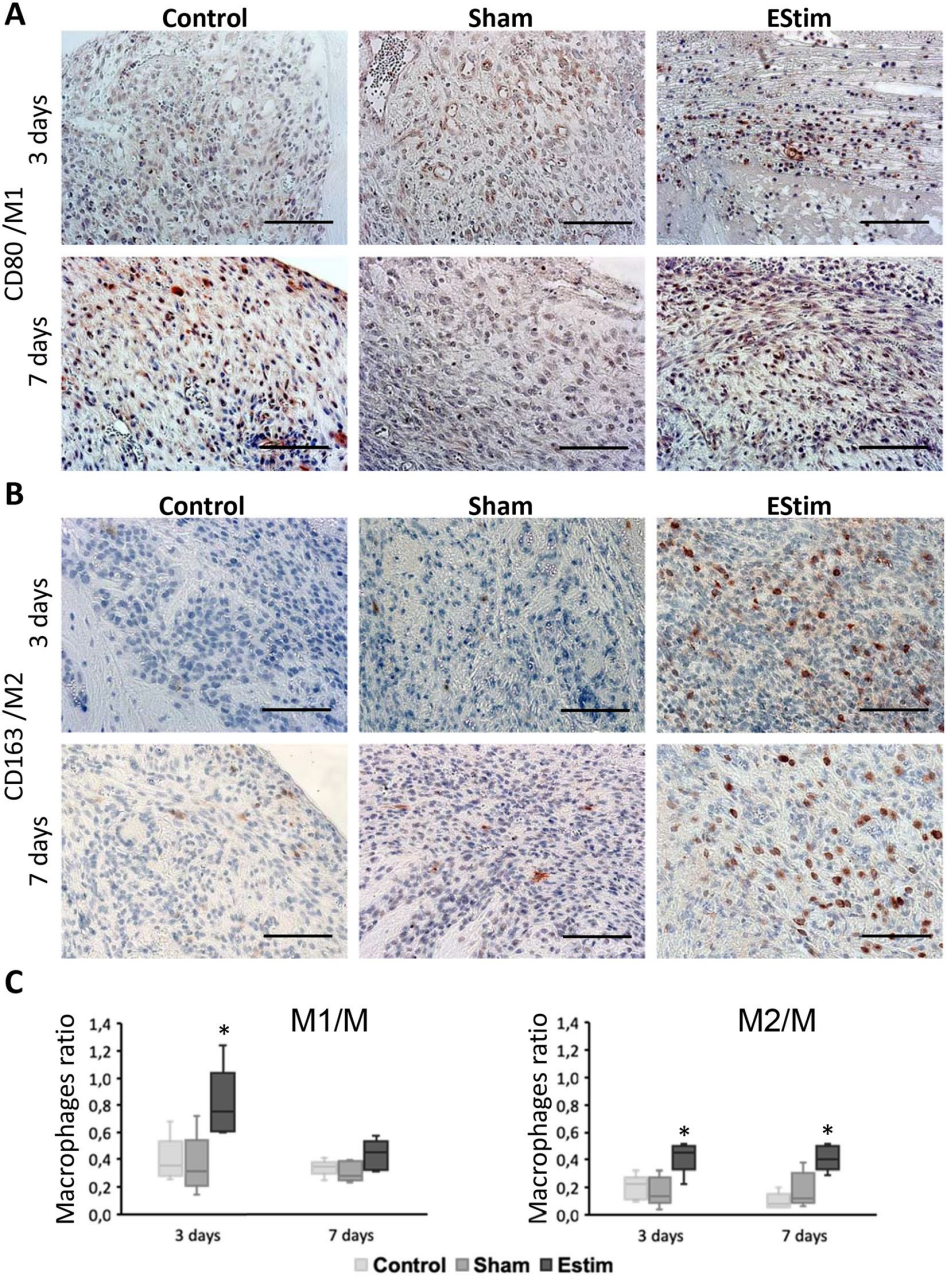
Immunohistochemistry analysis of CD80 (marker for M1 macrophages) and CD163 (marker for M2 macrophages) in limb stumps at 3 and 7 days post-amputation. **(A)** Representative images of immunohistochemical staining showing higher incidence of M1 macrophages at day 3 in EStim treated tissues (Scale bar = 100 µm). (**B)** Representative images showing higher incidence of M2 macrophages at days 3 and 7 in EStim treated tissues (Scale bar = 100 µm). (**C)** Ratio of M1 and M2 macrophages (normalized to M macrophages) at the distal end of the stump for all groups tested at 3 and 7 days post-amputation; *p < 0.05.

Immunohistochemical analysis of M1, M2 and M macrophages at early time points (3 and 7 days) after amputation indicated that EStim impacts the distribution, density and the constitution of macrophage populations in the distal area of the limb stump (Figs 2 and S1). Macrophages were distributed heterogeneously across the entire distal area of the stump tissue. In EStim treated animals more positively stained cells were detected in tissues adjacent to the bone, on the right and left sides, and in the bone marrow cavity. When different types of macrophages were considered within the same sample, all types were distributed similarly, however, the number of CD68 positive cells (M) was, as expected, higher than CD80 (M1) and CD163 (M2) positive cells (Fig. 2A,B).

While EStim exposure did not affect the number of CD68 positive cells (M) at the 3- and 7-day time point measurements (Supplementary Fig. S1), it did change the ratio between M1 and M2 macrophage populations. The number of M1 (pro-inflammatory) macrophages was significantly (p < 0.05) higher in EStim treated animals at day 3, and the number of M2 (anti-inflammatory) cells was significantly (p < 0.05) elevated at both 3 and 7 days. While the number of M1 and M2 macrophages remained the same between the 3- and 7-day measurements in control and sham treated animals, in EStim treated animals the number of M1, but not M2 cells, decreased significantly (p < 0.05) from days 3 to 7 (Fig. 2C).

### Cell proliferation

**Figure 3:**
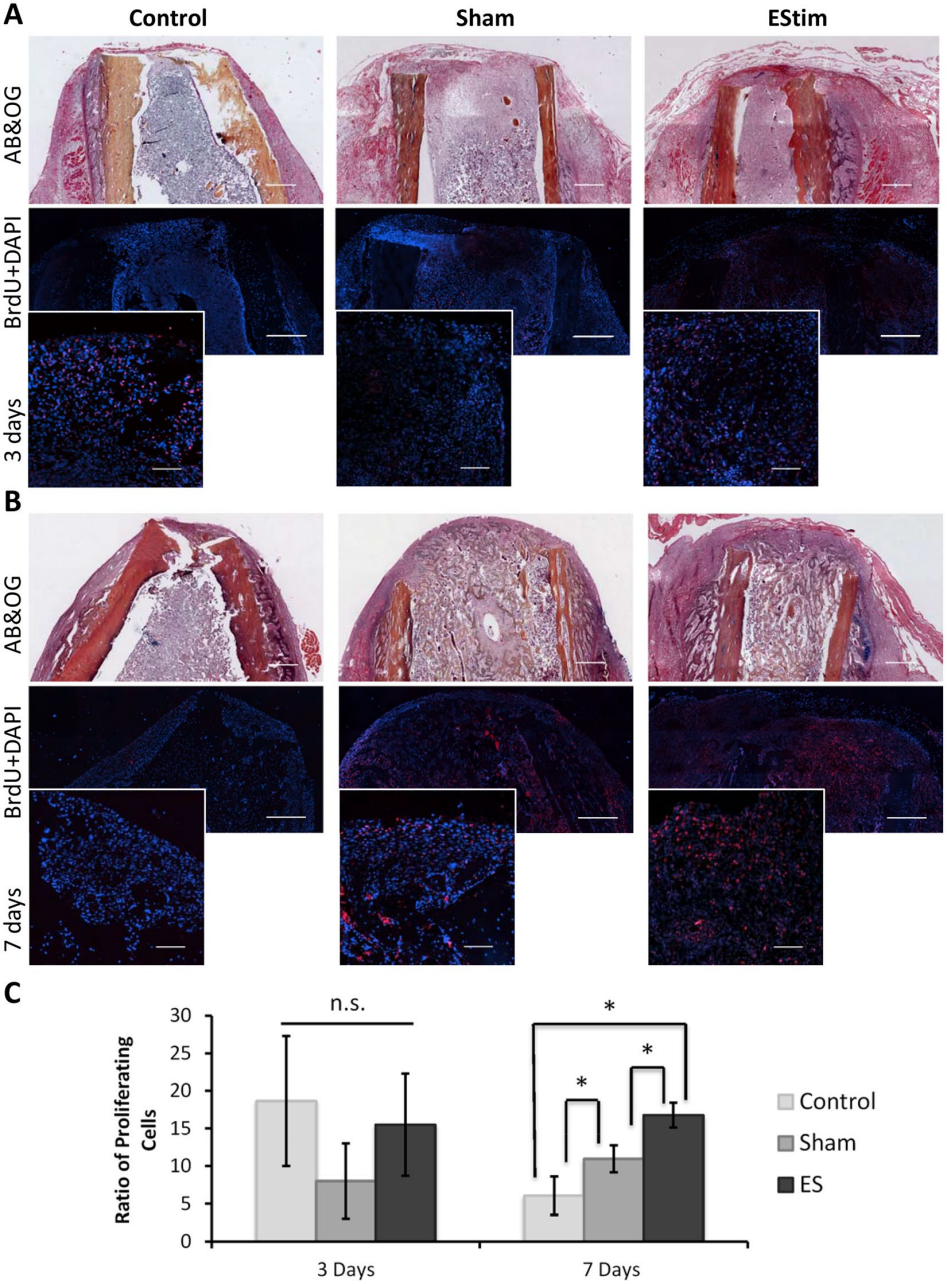
BrdU analysis of cell proliferation in the distal end of the stump in control, sham and EStim treated samples. (**A)** Top and middle rows represent overview of day 3 post-amputation control, sham and EStim stump sections stained with AB&OG and anti-BrdU antibody (red) counterstained with DAPI (blue) respectively (4×; Scale bar = 500 µm). Bottom row shows high magnification images of BrdU stainings (20×; Scale bar = 50 µm). (**B**) Representative images of stump sections from control, sham and EStim groups 7 days post-amputation. Top and middle rows show an overview of samples stained with AB&OG and anti-BrdU antibody (red) and counterstained with DAPI (blue) respectively (4×; Scale bar = 500 µm). Bottom row displays high magnification images (20×; Scale bar = 100 µm); (**C)** Ratio of proliferative cells measured at the distal end of the stump at days 3 and 7 post-amputation. n.s. - non significant (p ≥ 0.05); *p < 0.05.

Analysis of stained tissues revealed numerous BrdU-positive cells in the stump tissues, with a higher number of proliferating cells at the distal end of the stumps. Positively stained cells were evenly distributed among groups at day 3, with notably higher concentrations of stained cells in the area of the bone marrow (Fig. 3A,C). At day 7 positively stained cells were evenly distributed in the stump tissues of sham and control animals, whereas in EStim treated animals, cells were concentrated in the area surrounding the EStim device electrodes (Fig. 3B). No difference in the number of proliferating cells was observed between EStim treated and non-treated animals 3 days after amputation (Fig. 3C). However, at day 7, the number of proliferative cells was significantly (p < 0.05) higher in EStim treated stumps, compared to controls and sham (Fig. 3C). Between days 3 and 7 there was a significant decrease in the number of proliferating cells in the control samples, and a slight increase in the sham samples, while there was no change in the EStim treated stumps.

### New vessel growth

**Figure 4:**
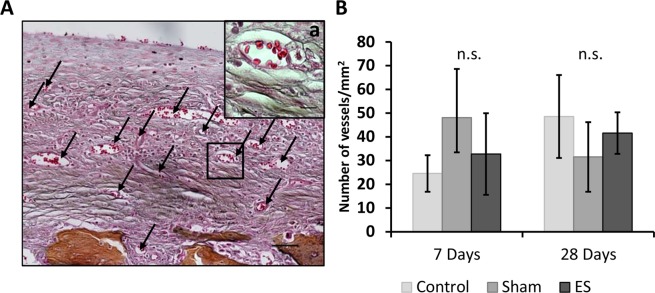
New vessel growth in the distal area of the EStim rat limb stumps 28 days post amputation. (**A)** Representative image displaying vascular ingrowth in the distal end of the EStim stump at day 28 post-amputation. (AB&OG staining; Scale bar = 100 µm). a – High magnification (40×; Scale bar = 10 µm) of new vessel. (**B**) Graph indicating density of new vessels at the distal end of the stumps in all 3 groups 7- and 28- days post amputation. n.s. - non significant (p ≥ 0.05).

Manual count of new vessels formed at days 7 and 28 in the distal area of the stumps revealed similar values in the 3 groups (Fig. 4).

### Gene expression

**Figure 5:**
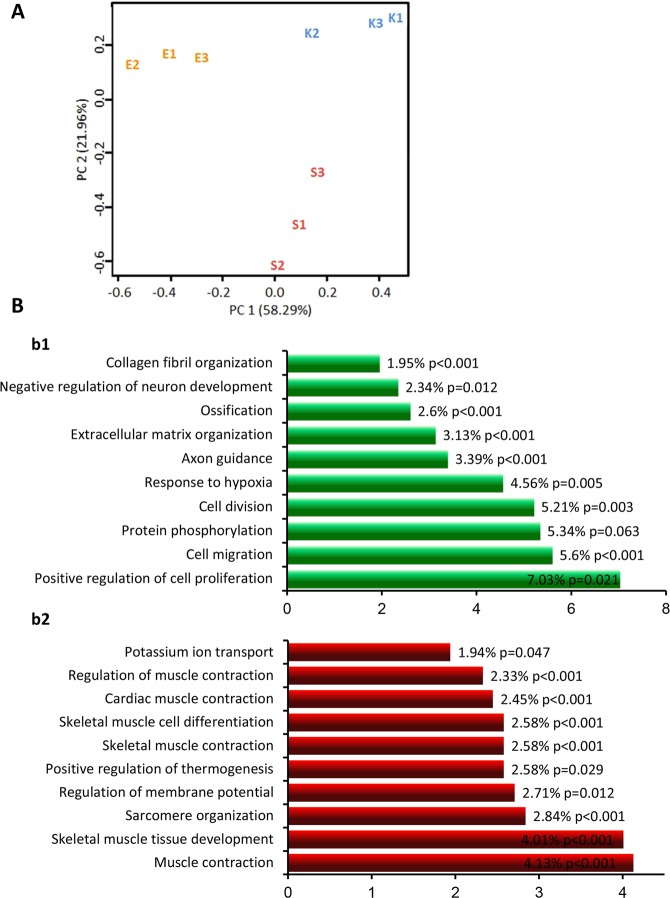
mRNA expression profiles in EStim treated limb stumps and non-treated controls (control and sham) are distinct. (**A)** Principle component analysis of normalized read counts showed strong effect of EStim treatment. Biological replicates for EStim (E1, E2, E3), sham (S1, S2, S3) and control (K1, K2, K3) samples are shown. (**B)** Top-10 significantly enriched biological processes in EStim and sham transcriptomes (FunReach analysis). Each the EStim and sham transcriptomes were tested against *Rattus Norvegicus* UniProt database, the top 10 most highly represented biological processes and the percentage of proteins predicted to belong to each category are shown for EStim (b1) and sham (b2) samples. The numbers shown at the right side of each bar indicated the % of genes (proteins) involved in each term and p-Value.

To characterize EStim induced molecular changes we performed deep RNA sequencing comparing transcriptomes in the 3 groups of limb stump tissues. Three biological replicates were analyzed for each condition and between 25 mln and 60 mln reads were generated for each sample (Supplementary Table S1). Normalized counts were used in principal-component analysis (PCA) for each biological replicate, and we found that sham- and EStim treated stump tissues differed greatly from the control samples (Fig. 5A). The number of differentially expressed genes (DEGs) was determined after multiple tests corrected the p value. The Q value was set at a false discovery rate (FDR) of 0.05, and differentially expressed genes were considered to be those transcripts passing this FDR. When comparing sham versus control tissues, there were a total of 406 differentially expressed genes. This number was dramatically increased when comparing the EStim versus the sham tissues; with a total of 1284 differentially expressed genes (all differentially expressed transcripts are provided in Supplementary Tables S1 and S2, representative examples are shown in Table 1).

**Table 1:** Examples of genes up- regulated in EStim.

*Gene*	Gene name	Log^2^ Fold change	*p*-value
*Mmp13*	Matrix Metalloproteinase 13 (Collagenase 3)	3.422626	5.08E-34
*Vsig4*	V-Set and Immunoglobulin Domain-Containing Protein 4	3.147901	1.12E-11
*Sox10*	SRY-Box 10	2.856724	4.24E-11
*Pla2g5*	Phospholipase A2 Group V	2.507993	1.45E-10
*Lif*	Leukemia Inhibitory Factor	2.406112	1.94E-07
*Mmp9*	Matrix Metalloproteinase-9	2.254128	5.13E-10
*Mmp3*	Matrix Metalloproteinase 3	2.170442	6.06E-10
*Shroom2*	Shroom Family Member 2, Apical-Like Protein	2.007132	3.83E-09
*Foxc2*	Forkhead Box C2 (MFH-1, Mesenchyme Forkhead 1)	1.962337	2.32E-05
*Dhh*	Desert Hedgehog	1.794296	3.23E-06
*Gsc*	Homeobox Protein Goosecoid	1.761111	0.000539
*Lhx2*	LIM Homeobox 2	1.736685	0.000146
*Dlx3*	Distal-Less Homeobox 3	1.734016	1.15E-06
*Wnt7b*	Wnt Family Member 7B	1.588366	8.13E-05
*Apln*	Apelin	1.504759	7.07E-06
*Lbh*	Limb Bud and Heart Development	1.476744	1.40E-07
*Hoxb2*	Homeobox B2	1.382072	0.000284
*Nrarp*	Notch-regulated ankyrin repeat protein	1.374706	0.00047
*Meox1*	Mesenchyme Homeobox 1	1.371066	1.35E-06
*Gli1*	Glioma-Associated Oncogene Homolog 1	1.307916	4.02E-06
*Wnt5a*	Wnt Family Member 5A	1.3033	7.28E-06
*Wnt5b*	Wnt Family Member 5B	1.214129	0.000162
*Mki67*	Marker of Proliferation Ki-67	0.985305	0.000111

For the subsequent analysis we focused on comparing the EStim and sham datasets.

**Figure 6:**
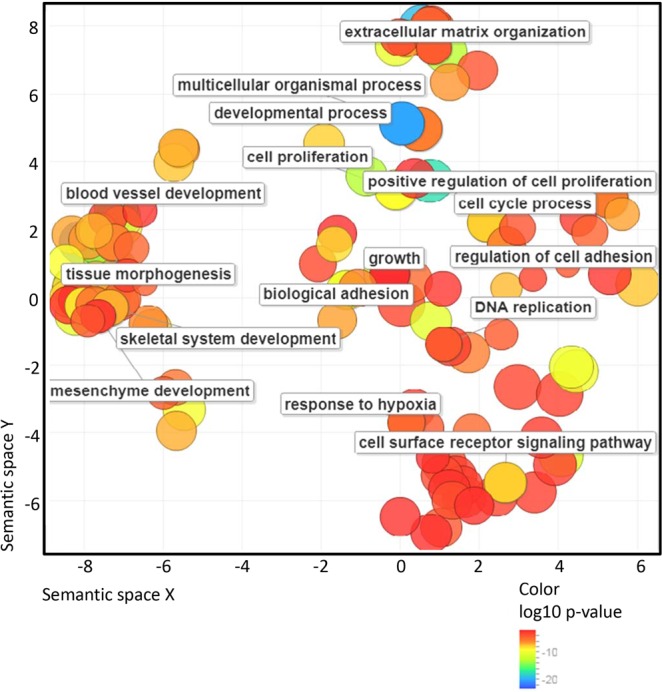
Gene Ontology (GO) analysis of up-regulated in EStim genes using REVIGO. The scatter plot shows the cluster representatives (terms remaining after reducing redundancy) in a two-dimensional space derived by applying multi-dimensional scaling to a matrix of GO terms semantic similarities. Bubble color indicates the p-value (legend in lower right-hand corner).

To reveal the types of processes regulated by EStim, we grouped the enriched pathways considering “large-scale functions.” Most of the transcripts up-regulated by EStim, were related to anatomical structure morphogenesis and development, animal organ and tissue development, extracellular matrix organization, cell differentiation, mitosis and migration (Figs 5B, 6 and Table S2). Most of the transcripts down-regulated by EStim were related to cell energy and metabolisms as well as muscle system process, muscle contraction and muscle structure development (Figs 5B, 6 and Supplementary Table S2).

Biological processes enrichment analysis against the UniProt database revealed that genes, significantly (p < 0.05) upregulated in EStim treated stump tissue, are involved in positive regulation of cell proliferation, negative regulation of apoptosis, cell migration, cell division, ECM organization, ossification, axon guidance and others (Fig. 5B). Conversely, among downregulated genes in EStim treated stumps, enrichment was shown for those involved in muscle contraction, skeletal muscle development, regulation of membrane potential, skeletal muscle differentiation, glycogen metabolic process, glycogen biosynthesis and others (Figs 5B, 6 and Supplementary Tables S1, S2). Thus, our gene expression analysis revealed profound transcriptional remodeling of EStim treated stump tissues and suggests numerous pathway targets for subsequent study and therapeutic interventions.

## Discussion

Limb amputation in mammals triggers a series of events that ultimately result in healing and fibrotic scar formation (reviewed in^12^); with some exceptions such as mouse digit tip amputation and, human fingertip which under special conditions have been shown to regenerate.

In a previous study, in an amputated rat forelimb model, we demonstrated that instead of eliciting scar formation, direct current EStim stimulated a regenerative response resulting in new bone, cartilage and vessel formation^32^. Regenerative response to amputation is strongly dependent on a tightly controlled inflammatory response, direct recruitment and proliferation of resident stem cells and regulation of biochemical, biophysical and biomechanical signals between cells and the ECM (reviewed in^33^). To evaluate if EStim is able to shift the injury response from healing/scarring toward regeneration, we designed this *in vivo* study to assess how EStim affects extracellular matrix deposition, macrophage response, cell proliferation and gene expression, all cellular functions known to play an important role in both of these processes.

Following injury in species capable of regenerating limbs, the ECM in local remaining tissues is broken down and replaced with a new regeneration-specific ECM^34^. Differences in matrix metalloproteinases (*Mmps*), responsible for ECM remodeling have been shown in regeneration-competent *Axolotl* and regeneration-deficient *Xenopus* froglets^35^. Of note, mammal and amphibian myoblasts responded similarly when stimulated with ECM substrates, suggesting that the key to inciting regenerative repair may be preserved in these species^36^. For most injuries, fibroblasts and myofibroblasts interact to produce extracellular matrix, mainly in the form of collagen, which ultimately forms the bulk of a mature scar^37^. In a skin wound model, Whitby DJ, and Ferguson MW described scar tissue in wound beds of adult mice as being covered by collagen fibrils, laid in parallel dense bundles. In contrast in fetus’, they found that collagen fibrils were deposited in a reticular fashion, almost identical to the surrounding dermis, and healing was scar-free^38^. In our electrically stimulated stump tissues, the ECM structure appeared to have less condensed fibrils, forming a net-like structure, similar to the reticular structure of fetus ECM. Conversely, in sham and control stump tissues, not exposed to EStim, the ECM was densely packed and appeared more like that seen in scar tissue. During healing, histolysis, in which ECM is degraded to release dermal fibroblasts, is mainly mediated by Mmps^39^. In regenerative injury response, proteinases are activated to degrade ECM and release cells for regeneration; *Mmp-8*, *-9*, and -10 are found to be up-regulated throughout blastema formation and *Mmp-13* is increased in stage 2 of epidermal wound closure (histolysis/dedifferentiation)^35^. These observations coincided with what we saw in our EStim treated stump tissues, that had elevated expression of various matrix metalloproteinase genes (*Mmp3*, *Mmp9* and *Mmp13*) and genes associated with ECM remodeling. While little is known about how EStim effects collagen deposition and alignment *in vivo*, *in vitro* studies have shown that electrical fields can effect extracellular protein structures^40^.

There are many reports in the literature describing the important role that macrophages play in tissue regeneration and wound healing. In experiments in which macrophages were depleted in axolotl and mice models both healing and regeneration were compromised^41–44^. In response to injury, M1 (pro-inflammatory) and M2 (anti-inflammatory) macrophages are regulated differently in healing versus regeneration; while M1 and M2 peak at the same time during regeneration, in non-regenerative tissues M1 response comes first followed by the rise of M2^9,10,43^. In the present study we saw a simultaneous increase in the ratio of M1 and M2 in electrically stimulated stump tissue at day 3 post amputation, then a sustained higher ratio in M2 macrophages at day 7. Our transcriptome data supported these observations in that the expression of *Vsig4* gene, responsible for M1 inhibition^45^ and *Pla2g5*, involved in M2 development^46^, were significantly increased in EStim treated stumps. In *in vitro* experiments, others have demonstrated that externally applied EStim affects macrophage migration, phenotype and plasticity^28,47,48^. However, little is known about the effect EStim has on macrophages *in vivo*. McLeand and Verge showed that exposing nerves to electrical stimulation shifted macrophage phenotype from M1 (pro-inflammatory) toward predominantly M2 (anti-inflammatory) in demyelinated nerves^29^.

In amphibian regeneration, interactions between immune- and stem cells have been shown to play an important role^43^. Stem cell migration and proliferation are essential in blastema formation and limb regeneration^15,18^. Our results showed that in addition to increasing the M2 population, EStim treatment also increased the rate of cell proliferation in stump tissue at day 7. These findings were supported by gene expression analysis that showed an upregulation of several proliferation marker genes (*mki67*, *Prr11*, *Fam111a*, *Mybl2*, *Mcm10*, *E2f8*, *Asf1b*, *Recql4*, *Cdh1 etc*.) in EStim samples. In fact, we found that the most enriched set of genes were those associated with cell proliferation. While, in this study we did not focus on identifying the origin of the proliferating cells in the distal stump tissues, we can speculate that they could have come from bone marrow derived MSC. This speculation is supported by reports in previous literature and our own data, that show the concentration of BrdU-labeled cells located in the bone marrow niche of EStim treated stumps and activation of ossification gene expression associated with EStim exposure^24–26,30,31,49–52^. The mechanisms by which EStim affect MSC proliferation are not known, however its involvement in cytoskeletal components, cellular microenvironment, ions and small molecular transport^53^ are the focus of several investigations. Our findings here and those of others, showing important changes in gene expression related to membrane potential, potassium ion transport and activity of sodium ion trans-membrane transporters, support the hypothesis that EStim acts by regulating ion and molecule transport through the cell membrane^21,27,54–56^. The observed increase of BrdU-positive stained cells in sham-treated stumps could have been caused by mechanical stimulation due to the presence of the metallic electrode (inactive device) in the bone marrow cavity, as MSCs and other cells have been shown to be highly mechanosensitive^57^.

In regeneration there is an initial increase in local stem cell populations and ECM remodeling, followed by new vessel growth^15^. The new vessels are crucial for the nourishment and waste removal required in the important growth of newly formed tissues characteristic in regeneration^8^. In a previous study we demonstrated that EStim increased new vessel formation in the distal end of limb stumps^32^. In the present experiments we found that vessel density in EStim treated stump tissue was higher than the controls, however not significantly. The limited number of samples in these studies could be the reason for this variation. Despite this, our RNA profile analysis showed up-regulation of the vascularization-controlling genes (*Foxc2*, *Apln*, *Nrarp*, *Shroom2)* in EStim treated samples.

From the time of injury until the successful healing/scarring or regeneration, surviving cells display a diversity of behaviors, including; death, growth, dedifferentiation, adhesion, communication, migration, mitosis, extracellular matrix remodeling, and differentiation^1^, all of which correlate with changes in transcription^58^. We observed important differences in gene expression profiles between EStim treated and non-treated stump tissues. In our EStim treated stump tissues Wnt signaling pathway members (*wnt5a*, *wnt5b* and *wnt7b)*, Homeobox (*Hox*) genes (*Gsc*, *Lhx2*, *Hoxb2*, *Meox1*) and Hedgehog pathway genes (*Gli1* and *Dhh*), were up-regulated. It is known that in axolotl limb regeneration as well as in mammal limb development, Wnt, Hox and other signaling pathways play an important role by forming expression gradients involved in determining axial identity^59^. Wnt signaling has been shown to have important functions during mammalian limb bud initiation, limb outgrowth, early limb patterning, and later in limb morphogenesis events^60^. Hox genes have been reported to encode homeodomain-containing transcription factors that determine cell and tissue identities in the embryo during development and are very conservative in evolution that justify the importance of their role in development^61^. Hedgehog pathway genes (*Gli1* and *Dhh*), also activated in our EStim treated stump tissues, are known to encode signaling molecules that play a role in morphogenesis regulation^62,63^. Moreover, a number of genes involved in cell cycle regulation, stem cell differentiation and proliferation were activated in EStim treated tissues. The majority of genes, down-regulated in EStim treated tissues, were those involved in cell energy metabolism as well as skeletal muscle homeostasis, that correlate with findings reported in axolotl regeneration. It was shown that Axolotl blastema cells actively repress a muscle program by suppressing genes similar to those we observed to be suppressed^64^ in our tissue samples. This suggests that EStim activates/suppresses defined pathways and thus shifts tissue injury response from one characteristic of healing/scarring toward that seen in development and/or regeneration.

Regeneration is known to be time dependent, regulated by mechanisms coordinating transcription within and across cell types at different stages of appendage regeneration^65^. While in this study we did not focus on how EStim affects temporal and positional expression gradients, this is an important goal in our ongoing and future studies in order to answer the question how EStim acts to create a pro-regenerative environment.

Despite the promising results obtained in the present experiments, it is important to consider some limitations. First, in order to reproduce the model Becker used in his 1972 study, animals were operated at the same young age. It is well known that wound healing ability declines with age^66,67^, therefore in future studies it will be important to compare the observed effects EStim has on healing in aged animals. Second, due to several technical limitations related to the limb amputation model and ethical concerns (such as bleeding, self-mutilation, infection, dislodging of the EStim device, and additional animal suffering) in the present study skin was sutured over the stump after surgery. Aware that this could negatively impact the regenerative response^68^ skin was excluded from this analysis. Third, the effect EStim has on macrophage polarization was analyzed by means of M1- and M2- marker staining. Markers used in this study are widely recognized for their distinct affinities to specific proteins present in M1 and M2 cells, still the possible presence of intermediately polarized M1/M2 cells^69^ could have interfered with our results.

In summary, our data supports the hypothesis that EStim shifts injury response, in our amputated rat limb model, from healing/scarring toward regeneration. This effect appears to be in the early inflammatory response where it stimulates cell proliferation and ECM remodeling. EStim induces profound changes in gene expression, including activation of genes involved in morphogenesis and development. Additional studies are needed to spatially and temporally characterize this tissue reaction to electricity and to elucidate which cellular players orchestrate it. A greater understanding of these underlying mechanisms will bring us closer to the possibility of being able to control cellular functions, which could later be translated into new treatments that promote regenerative responses.

## Material and Methods

All animal experiments were performed in accordance with guidelines established by our animal care and oversight committee at the Johann Wolfgang Goethe-University in Frankfurt am Main, Germany and were approved by the Veterinary Department of the Regional Council in Darmstadt, Germany (Regierungspräsidium Darmstadt, Veterinärdezernat, Wilhelminenstraße 1–3) (Project No. FU/1114).

To assess the effects of EStim on limb tissue regeneration, the right forelimbs of 60 male Sprague Dawley rats (Charles River Labs Int., Germany) (age = 5 weeks; weight = 100–150 g) were amputated and the limb stumps were treated with: 1) Electrical stimulation (implanted functioning EStim device) (n = 20), 2) No electrical stimulation (implanted deactivated device – sham control) (n = 20), and 3) No electrical stimulation (no device – control) (n = 20). Table 2 shows the treatment, the number and the distribution of animals in their respective groups.

**Table 2:** Treatment, number and distribution of animals into groups.

Group	Treatment	Time point (days)	Number of animals	Analysis
Control	No	3	5	Histology, IHC
		7	10	Histology, IHC, deep RNA-seq
		28	5	Histology, IHC
Sham	Disabled device	3	5	Histology, IHC
		7	10	Histology, IHC, deep RNA-seq
		28	5	Histology, IHC
EStim	Active device	3	5	Histology, IHC
		7	10	Histology, IHC, deep RNA-seq
		28	5	Histology, IHC

### Limb amputation and EStim device implantation surgeries

Electrical stimulation was applied using a purpose-built bimetallic device and surgeries were performed as described previously^32^. Animals were euthanized (CO_2_ inhalation) at 3-, 7- and 28- days post amputation, weighed, and the limb stumps were collected and carefully examined macro- and microscopically. In the macroscopic examination, the skin covering the limb stumps of the EStim and sham-treated animals was carefully incised and the EStim device and corresponding electrodes were controlled for integrity and carefully removed. In addition, all limbs were inspected for signs of infection and/or tumors. The specimens were fixed in Zinc-Formal-Fixx, (Thermo Electron, USA) for 24hrs and stored for subsequent histomorphometric and immunohistological analysis or stored in liquid nitrogen for subsequent RNA isolation.

### Tissue preparation

Fixed samples were decalcified in 10% EDTA/TRIS-HCl (pH 7.4) for 14 days and were embedded in paraffin. Longitudinal sections (5 µm thick) were taken parallel to the long axis of the humerus for all samples from all groups and timepoints. Sections were selected based on the following criteria; presence of stump tissue from side to side, absence of damaged tissues, presence of humerus bone’s medullary cavity and compact bone on both sides of the cavity (Supplementary Fig. S2). Selected sections were stained according to the specific parameters analyzed, as described below. Analysis and quantitative evaluations were performed using light and fluorescent microscopy (Ti-E, Nikon GmbH, Germany) and image analysis software (NIS-Elements 4.4, Nikon GmbH, Germany). For all staining and analysis, a minimum of 3 sections per limb, and 5 limbs per groups for each time point, were used.

### ECM collagen deposition measurements

An important distinguishing characteristic between healing/scarring and regeneration tissue is the content and distribution of collagen fibers. In order to measure these morphological structures in the limb stumps, tissue sections were deparaffinized and hydrated, colored with Picro Sirius Red (0.1% of Sirius Red in saturated aqueous picric acid) (Sigma-Aldrich, Germany) for 1 hour, washed two times in 0.5% acetic acid solution, dehydrated in three changes of 100% ethanol, cleared in xylene and mounted. Images were visualized using fluorescence microscopy (Ti-E, Nikon GmbH, Germany)^70^. To evaluate the distribution of collagen fibrils, the orientation (anisotropy), and the distance between fibrils (condensation), image analysis software (ImageJ; NIS-Elements 4.4, Nikon GmbH, Germany) was used. A total of three sections per animal, and five samples per group/timepoint were analyzed. To quantify the parallel distribution of collagen fibers (anisotropy), “FibrilTool” plug-in for ImageJ software was used as described by^71^. Anisotropy was measured in a region of interest (ROI) measuring 250 µm × 250 µm, located at the distal end of the stump in the middle of medular cavity (Supplementary Fig. S2), and the mean value for 5 animals was calculated and used to represent each group/time point. The distance between fibrils (condensation) was measured in 20 randomly chosen locations within the same ROI, and the mean values for each animal was calculated. These values were used to calculate the mean value for 5 animals, to represent each group/time.

### Macrophage number and distribution measurements

To quantify different macrophage phenotypes present in the rat limb stumps, tissue sections were stained with primary specific antibodies against (1) general and non-activated macrophages (M - CD68 marker; MCA341GA; BIO-RAD Laboratories; GmbH Germany), (2) pro-inflammatory macrophages (M1 - CD80 marker; AA270-321; Antibodies online GmbH, Germany), and (3) anti-inflammatory macrophages (M2 - CD163 marker; MCA342R; BIO-RAD Laboratories GmbH, Germany). Briefly, tissue sections were deparaffinized, rehydrated and trypsin (CD68 and CD163 antibodies) or heat (CD 80 antibody, Citrate buffer pH6) antigen retrieval was performed before staining with antibodies. For signal detection, an EnVision + System-HRP (AEC) kit (Dako, Germany) was applied for sections stained with CD163 and CD68 antibodies or an EXPOSE Rabbit HRP-AEC detection IHC Kit (Abcam, Germany) was used for sections stained with CD80 antibody. Finally, a counterstain with hematoxylin was performed. An Isotype identical (IgG1) non-specific mouse antibody served as a negative control (eBioscience, Germany). Three slides per animal were analyzed using light microscopy (at 10×) (Ti-E, Nikon GmbH, Germany) and image analysis software (NIS-Elements 4.4, Nikon GmbH, Germany). Five standardized regions of interest (ROIs – 420 µm × 315 µm) (at 20x magnification) were selected in the distal area of the stump. Stained macrophages were manually counted by two independent trained examiners, blinded to the group makeup, and similar results were obtained. After obtaining the number of CD80+ cells, the ratio of cells expressing M1 phenotype was obtained by dividing it by the number of CD68+ cells for each ROI. The same calculations were done to obtain the ratio of M2 macrophages (CD163+ cells/CD63+ cells). Mean values were obtained for each ROI and each animal, and were used to statistically analyze the differences among groups at each timepoint. The presence of the CD68 surface marker designates a macrophage phenotype, which is a common marker for both M1 and M2 states and other cells from monocyte lineage, therefore the sum of M1 and M2 is not necessarily equal to the number of CD68+ cells^72^.

### Cell proliferation measurements

Cell proliferation was assessed by immunohistochemical detection of incorporated Bromdesoxyuridin (BrdU) at the distal end of the limb stumps. Animals received a peritoneal injection of BrdU labeling reagent (Invitrogen, eBiosciences, Germany) 1 day prior to being euthanized. After sample processing, as described above, paraffin sections were deparaffinized and treated with 2 M HCl solution at 37 °C for 20 min and 0.05% trypsin for 30 min at 37 °C. Endogenous peroxidase activity was blocked by 3% H_2_O_2_ solution, after which 7% goat serum, 1% BSA in TBS buffer blocking solution was applied for 1 hour. Staining was performed at +4 °C overnight with primary anti-BrdU antibody (MoBU-1, BioLegend, Germany, 1:100). Secondary antibodies (ab150128, Abcam, Germany, 1:500) were applied for 1 hour at room temperature and tissue sections were covered with DAPI-containing Fluoroshield Mounting Medium (ab104139, Abcam, Germany). For each group, tissue sections from three animals were analyzed under a fluorescence microscope using *NIS* software. *Quantification:* Three regions of interest (ROI) located at the left, center, and right of the distal stumps (at 20x magnification) were selected. Positive BrdU- and DAPI-stained cells were thresholded, and for each ROI the area with BrdU-positive cells was normalized to the total (DAPI-stained) area of cells to obtain the fraction of proliferative cells in each ROI. The mean value of the 3 ROIs was calculated for each animal and the values of 3 animals per group were used for subsequent statistical analysis.

### New vessel growth measurements

Tissue sections used in the ECM measurements were also used for new vessel growth measurements, using bright field microscopy. Quantification of new vessels present in the distal stump tissue was performed on five samples from each group at days 7 and 28 post-amputation. Two trained examiners (blinded to the group composition) counted new vessels manually in defined ROIs, located in the distal area of the stump tissue, of five samples. The number of vessels was normalized to the dimensions of the evaluated area (ROI). The density of newly-formed vessels in each sample was obtained, mean values were calculated for each group and these were statistically analyzed.

### Gene expression measurements

#### Preparation and sequencing of RNA-seq libraries

Total RNA was extracted with Trizol reagent (Invitrogen) according to the manufacturers’ protocol from 3 samples from each group collected 7 days post-amputation. mRNA was purified with NEXTflex Poly(A) beads (Bioo Scientific). RNA libraries were prepared with NEXTflex® Rapid Directional qRNA-Seq™ Kit (Bioo Scientific) following the manufacturer’s protocol and sequenced on an Illumina HiSeq2500 machine.

#### Differential gene expression analysis

Illumina sequencing reads were mapped to the rat genome assembly Rnor_6.0 using STAR v.2.5.3 (https://www.ncbi.nlm.nih.gov/pubmed/23104886) and gene annotations from Ensembl release 90. Gene counts were generated by the “quantMode GeneCounts” option in STAR. Count normalization was performed with RUVSeq software^73^ with k = 4, and differential gene expression analysis was carried out with edgeR package^74^. Significantly differential expression genes were selected based on a corrected P-value of 0.05 as the threshold. GO term enrichment analysis of differentially expressed genes was performed with GOrilla^75^ and functional enrichment analysis against the UniProt database was performed with the FunReach analysis tool^76,77^. Revigo web server was used for visualization of enriched GO terms by means of a semantic similarity-based scatterplot^78^.

### Statistical analysis

For comparisons among and between groups, Kruskal-Wallis-Test with multiple Conover-Iman-Comparison (Bonferroni-Holm-correction) was applied on BiAS for WindowsTM version 11.0 software (http://www.bias-online.de). Data are presented as mean ± SD and a significance level was set at p < 0.05.

## Supplementary information

Supplementary figures S1 and S2

Supplementary Table S1

Supplementary Table S2

## Data Availability

The Illumina sequencing data were deposited to the NCBI Sequence Read Archieve under BioProject Accession Number PRJNA532925 (SRA entries SRR8902417-SRR8902425).
