# Supplementary material for: Electrical stimulation shifts healing/scarring towards regeneration in a rat limb amputation model
Source: Sci Rep. 2019 Aug 7;9:11433. doi: 10.1038/s41598-019-47389-w (PMC6685943; doi:10.1038/s41598-019-47389-w)
Supplement: Supplementary file 1 — Supplementary figures S1 and S2 [file 41598_2019_47389_MOESM1_ESM.pdf]

Title: Electrical stimulation shifts healing/scarring towards regeneration in a rat limb amputation model

Authors:

KMC Oliveira[1], JH Barker [1], E Berezikov[2], L Pindur[1,3], S Kynigopoulos[1], M Eischen-Loges[1], Z Han[1], MB Bhavsar [1], D Henrich[4], L Leppik[1]*

Affiliations:

1. Frankfurt Initiative for Regenerative Medicine, Experimental Orthopedics & Trauma Surgery, J.W. Goethe University, Frankfurt am Main, Germany

2. European Research Institute for the Biology of Ageing, University Medical Center Groningen, Groningen, The Netherlands.

3. Department of Plastic, Hand and Reconstructive Surgery, BG Trauma Center Frankfurt am Main gGmbH, Frankfurt am Main, Germany (current address).

4. Department of Trauma, Hand and Reconstructive Surgery, J.W. Goethe University, Frankfurt am Main, Germany

***Corresponding author:**

Dr. Liudmila Leppik

Email: Liudmila.Leppik@kgu.de

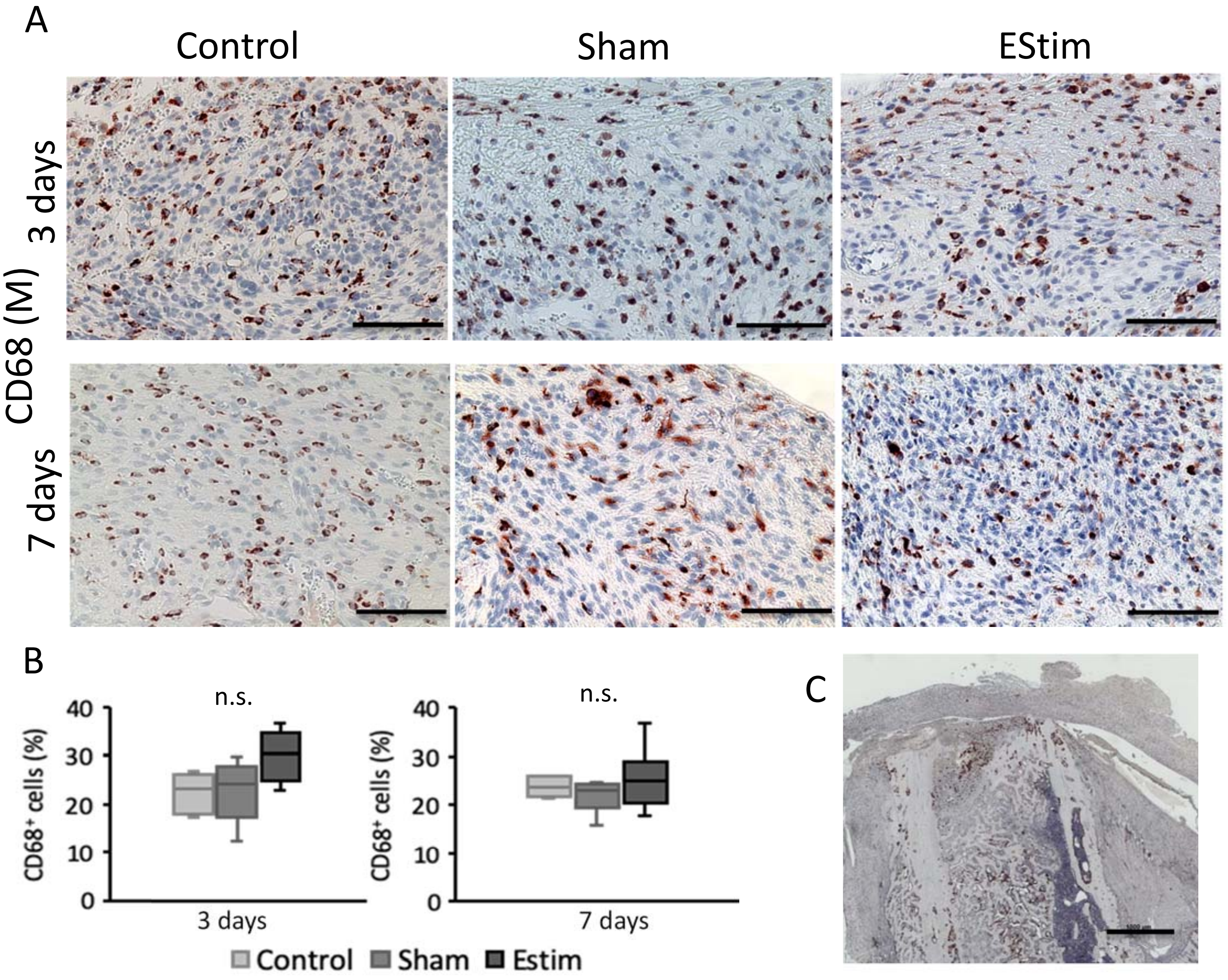

Supplementary Figure S1. Immunohistochemistry analysis of CD68 (pan-macrophage marker) in limb stumps at 3- and 7-days post amputation. **A:** Representative images of anti-CD68 staining in control, sham and EStim treated tissues at day 3 (top raw) and day 7 (bottom raw), (Scale bar = 100µm). **B:** Graph indicating ratio of CD68+ cells in the distal end of the stumps in all 3 groups 3 and 7 days post amputation. n.s. - non significant ($p \geq 0.05$). **C:** Representative image displaying CD68+ cells in the distal end of EStim treated stump, 7 days post amputation (Scale bar=1000µm).

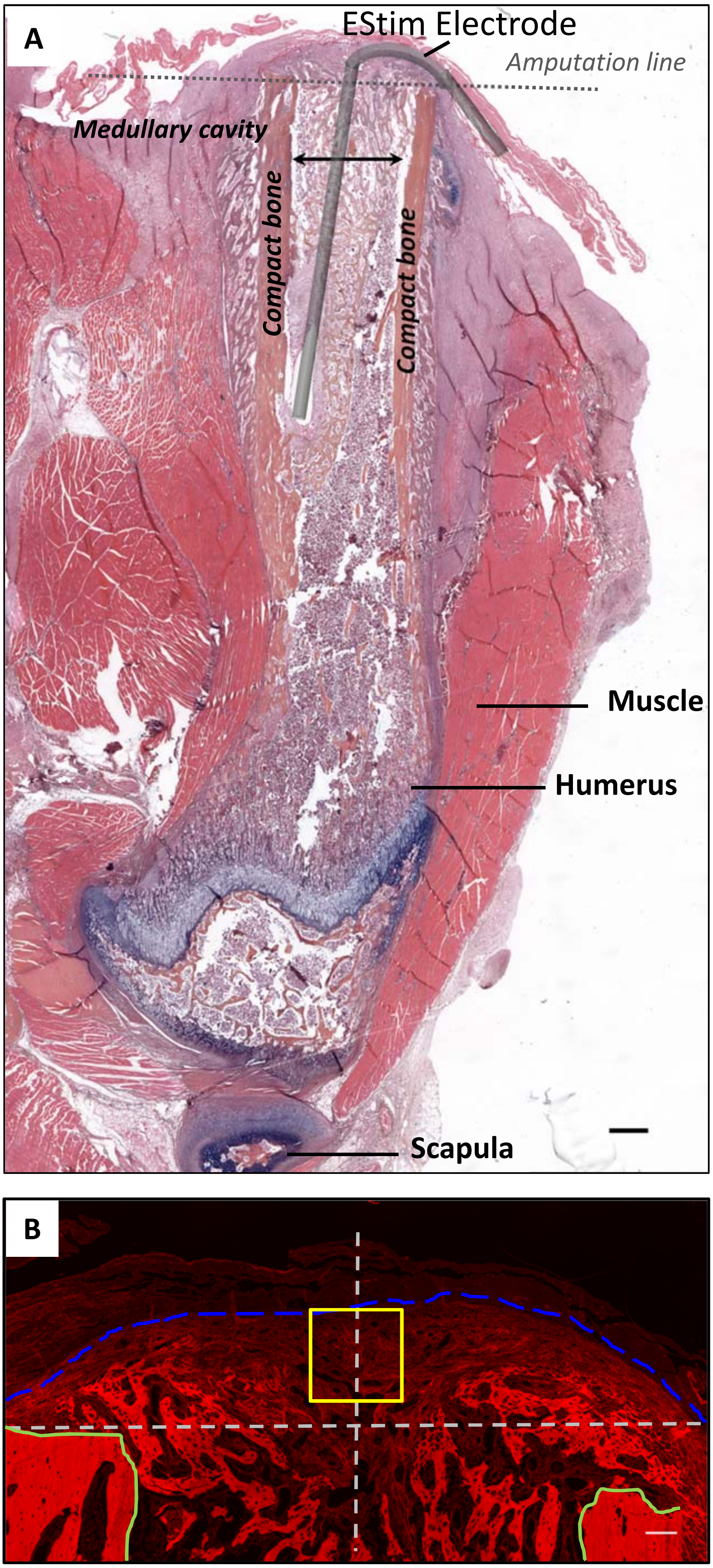

Supplementary Figure S2. Representative longitudinal section of the EStim stump 7 days post amputation. A: Longitudinal section showing stump anatomy, from side to side, of humerus with the presence of compact bone, medullary cavity, scapula, and muscle tissue. Position of EStim device electrode and amputation line (dotted line) indicated in the image (AB&OG staining, 4x magnification, Scale bar = 500µm). **B:** Representative high magnification image displaying the distal end of the EStim stump at day 7 post-amputation (blue line), position of the ROI for the ECM analysis (yellow square) relative to the distal end of the stump and the end of the humerus (light green lines) (Sirius red staining; 10x magnification; Scale bar = 100µm).
